# Impulsiveness levels among patients with medication-overuse headache accompanying chronic migraine or tension-type headache

**DOI:** 10.1055/s-0045-1807716

**Published:** 2025-05-13

**Authors:** Ruken Simsekoglu, Nestug Keskin, Tugba Cankay, Sumeyye Cakmak, Temel Tombul

**Affiliations:** 1 Istanbul Göztepe Prof. Dr. Suleyman Yalcın City Hospital, Department of Neurology, Istanbul, Turkey.; 2 University of Health Sciences, Istanbul Bakıroy Dr. Sadi Konuk Training and Research Hospital, Department of Emergency Medicine, Istanbul, Turkey.

**Keywords:** Migraine Disorders, Tension-Type Headache, Impulsive Behavior, Headache Disorders, Secondary

## Abstract

**Background:**

Impulsiveness in primary headaches is not well understood.

**Objective:**

To analyze impulsiveness in patients with medication-overuse headache (MOH) and either chronic migraines or chronic tension-type headaches (TTHs).

**Methods:**

This cross-sectional study included 119 participants (96 female) divided into 3 groups: the migraine with MOH (M-O, *n* = 44, age = 36.6 ± 11.1), the tension-type headache with MOH group (TTH-O, *n* = 38, age = 42.6 ± 11.8), and the healthy control group (HC, *n* = 37, age = 36.9 ± 13.1). The Barratt Impulsiveness Scale-11 Short Form (BIS-11 SF), Beck's Depression Inventory (BDI), Beck's Anxiety Inventory (BAI), and Pittsburgh Sleep Quality Index (PSQI) were used to assess impulsiveness, depression, anxiety, and sleep quality, respectively.

**Results:**

Impulsiveness levels were significantly higher in the M-O ( *p* < 0.01) and TTH-O ( *p* < 0.01) groups compared with the HC. However, no significant difference in impulsiveness was found between the M-O and TTH-O ( *p* > 0.05). The PSQI scores were significantly higher in the M-O and TTH-O compared with the HC ( *p* < 0.01). Additionally, anxiety scores were notably higher in the M-O compared with both the TTH-O and HC ( *p* < 0.01).

**Conclusion:**

The present study, which compared the M-O and TTH-O groups with HC in terms of impulsiveness, with no significant differences in parameters such as age, gender, schooling, frequency of headache attacks, and disease onset duration, concluded that both patient groups exhibited higher impulsiveness compared with the controls. Furthermore, the lack of difference in impulsiveness between MO and chronic TTH-O patients with a common denominator of MOH suggests that it may be associated with MOH, which is a shared subset of two distinct headache disorders.

## INTRODUCTION

Impulsiveness is a disorder characterized by acting without thinking or conscious judgment, responding quickly, making rapid decisions, and having a reduced focus on future plans. 1 It is encountered particularly in cognitive disorders where frontal executive functions are affected and serves as a supportive criterion in the diagnosis of many psychiatric illnesses. 2 In the Diagnostic and Statistical Manual of Mental Disorders, Fifth Edition (DSM-V), impulsiveness is defined as a dimension of disinhibition, characterized by sudden, momentary responses to stimuli, exhibiting behaviors that could harm oneself without thinking due to a sense of urgency, and reacting without considering the consequences. 3

Although impulsiveness is more prevalent in young people and male adults, its lifetime prevalence is around 16.9%. 4 Studies have shown that individuals with substance addiction often exhibit impulse control disorders. 5 Moreover, research suggests that medication-overuse headaches (MOHs) and substance addiction may share a similar pathophysiology. 6

Medication overuse is characterized by the regular intake of acetylsalicylic acid and nonsteroidal anti-inflammatory drugs (NSAIDs) for 15 days or more per month. It also includes the use of triptans, opioids, and ergot alkaloids for 10 days or more per month for acute or symptomatic relief of headaches. 6 7 Further, MOH is defined as a headache occurring on 15 or more days per month due to the regular and excessive use of acute or symptomatic headache medications for more than 3 months. 7

Although the prevalence of MOH is reported to be 1% in the general population, it can increase up to 70%, especially in primary headaches. 8 It can frequently coexist with primary headaches, particularly migraine and tension-type headaches (TTHs), making their treatment more challenging. 7 9 Several studies suggest that MOH, which exhibits an addictive pattern, may be associated with impulsiveness. 10 11

Migraine and TTH are two primary headache types that constitute a significant portion of neurology clinical practice. Clinical presentations and follow-ups are more frequent in migraines, which often causes more severe pain and significantly limits daily life activities during attacks. As a result, these patients are frequently studied when investigating the neurocognitive effects of primary headaches. In contrast, cognitive studies related to TTH are limited, and its relationship with cognitive impairment may be less specific due to factors such as the condition's high lifetime prevalence and its milder pain intensity. 12

The relationship between impulsiveness and chronic migraine with MOH has been studied several times but, to our knowledge, impulsiveness in chronic TTH with MOH (TTH-O) has not. 11 13 14 15 16 In this study, we aimed to examine patients with M-O and TTH-O in terms of impulsiveness and compare them with healthy controls.

## METHODS

The present prospective cross-sectional study included patients with MOH and either chronic migraine or TTH who presented to the headache outpatient clinic at Istanbul Göztepe Prof. Dr. Suleyman Yalcin City Hospital between January 2022 and January 2023. Ethical approval for the study was obtained from the Istanbul Göztepe Prof. Dr. Suleyman Yalcin City Hospital's Ethics Committee, under the decision number 2021/0648. All participants signed an informed consent form. Diagnoses were made in accordance with the third edition of the International Classification of Headache Disorders (ICHD-3). 7

The groups comprised patients with chronic migraine and MOH (M-O), patients with TTH and MOH (TTH-O), and healthy controls (HC). At our headache outpatient clinic, patients kept diaries to record the days they experienced headaches, as well as their intensity, duration, and how they resolved, enabling a more accurate follow-up. Information about the headaches was collected from these diaries and patient interviews. All cases of medication overuse were NSAIDs.

Patients suspected of having both migraine and TTH were excluded from the study. Those diagnosed with neurological or psychiatric diseases, drug abuse, alcoholism, and with additional diseases leading to analgesic use (e.g., discopathies, rheumatological diseases) were also excluded from the study.

The control group was selected from relatives of the study conductors, all of whom had no known diseases, no hospital admissions, and were matched with the patient groups in terms of age, schooling, and gender. The scales used in the study were administered by the attending physicians conducting the study. Tests were not performed during headache attacks.

**Figure 1:**
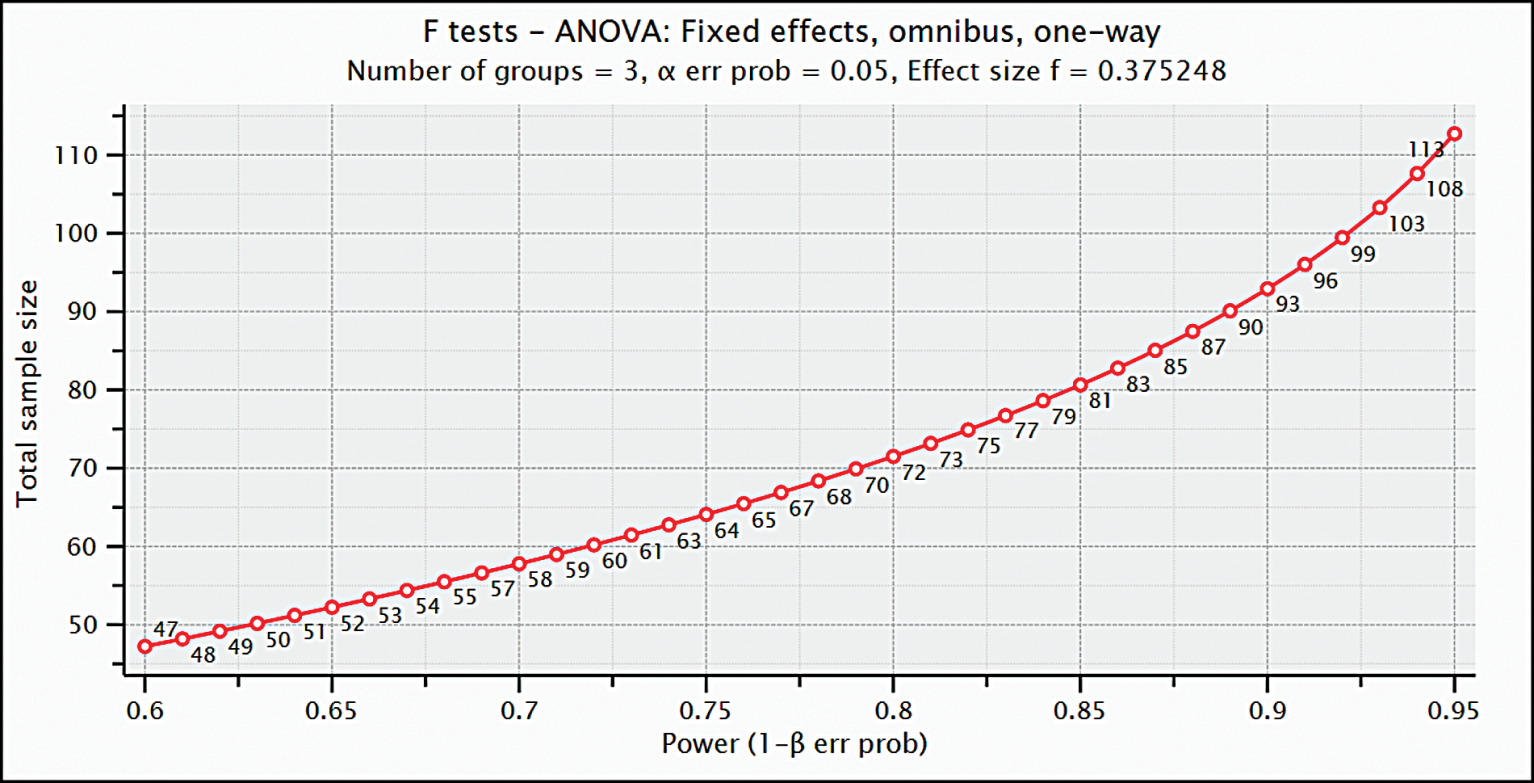
ANOVA test for sample size, power analysis.

At the outset of the study, a power analysis was conducted, based on the Barratt Impulsiveness Scale(BIS), determining that a total of 114 participants, with 38 individuals in each group, were required for the study, based on a 95% confidence level (CI, 1-α), a 95% test power (1-β), and an effect size of f = 0.375 ( Figure 1 ).

The age, sex, and years of schooling of the participants in each study group were recorded. The family history of primary headache (migraine or TTH), monthly headache attack frequencies, and the duration of time elapsed since the diagnosis were noted for the patients. The severity of headache attacks was scored between 1 and 10 points using the Visual Analogue Scale (VAS). The number of analgesic medications that the patients started using in the last 3 months was reported as medication use per month.

The BIS-11 short form (BIS-11 SF) was administered for impulsiveness. The BIS-11 SF is a shortened version consisting of 15 questions from the 30-item BIS. It includes scoring in three different categories: non-planning, and attentional and motor impulsiveness. Scoring is as follows: rarely = 1, sometimes = 2, often = 3, and always = 4. Questions 1, 5, 6, 11, and 15 are reverse-scored. The test yields a total score ranging from a minimum of 15 to a maximum of 60 points, without a cut-off value, and it is used in comparative studies. The Turkish version of BIS-11 SF was validated by Tamam et al. 17

The Pittsburgh Sleep Quality Index (PSQI) was used to investigate the effect of sleep on impulsiveness. It was developed by Buysse et al. in 1989, 18 and its Turkish validity and reliability study was conducted by Ağargün et al. 19 The Beck Depression Inventory (BDI) was used to assess the presence and severity of depressive symptoms, 20 its validity and reliability studies have been conducted for the Turkish population. 21 The Beck Anxiety Inventory (BAI) were used to measure the participants' levels of anxiety, 22 validity and reliability studies have been conducted by Ulusoy M for the Turkish version. 23

### Statistical analysis

Mean, standard deviation (SD), median, minimum, maximum value frequency, and percentages were utilized for descriptive statistics. The distribution of variables was checked using the Kolmogorov-Smirnov test. For quantitative data, we used the analysis of variance (ANOVA, the Tukey test), as well as the Kruskal-Wallis and Mann-Whitney U tests. The Chi-squared test was utilized for the comparative qualitative data. The IBM SPSS Statistics for Windows (IBM Corp., Armonk, NY, USA), version 27.0, was utilized for statistical analysis. A Spearman's correlation analysis was performed. A regression analysis was conducted to measure the causality between the BIS and its subdimensions and the variables in the M-O and TTH-O groups. The BIS and its subdimensions were evaluated as dependent variables in the regression models, through the backward method. Initially, all variables were included in the model, with the insignificant ones being removed one by one. The variables with the highest explanatory power for the BIS and its subdimensions remained.

## RESULTS

A total of 119 subjects were included in the study (96 female and 23 male). Out of 119 participants, 44 were in the M-O group, 38 were in the TTH-O group, and 37 were in the HC group. The average age was 38.6 ± 12.2 years, with a mean schooling duration of 10.19 ± 4.3 years. There were no considerable variances observed among the 3 groups in terms of age ( *p* = 0.21), sex, and years of schooling ( Table 1 ).

**Table 1:** Demographic and clinical characteristics of participants

Parameters		Groups			*p* -value
		Migraine (n = 44)	TTH (n = 38)	Control (n = 37)	
Age, years, mean ± SD		36.6 ± 11.1	42.6 ± 11.8	36.9 ± 13.1	0.058 ^+^
Sex, n (%)	Female	39 (88.6%)	30 (78.9%)	27 (73%)	0.195 ^&^
	Male	5 (11.4%)	8 (21.1%)	10 (27%)	
Schooling, years, mean ± SD		11.2 ± 4.1	10.4 ± 4.5	11 ± 4.3	0.747*
Family history, n (%)	Yes	38 (86.4%)	15 (39.5%)	−	< 0.01 ^&^
	No	6 (13.6%)	23 (60.5%)	−	
PSQI, mean ± SD		6.5 ± 4.9	8.0 ± 5.2	3.7 ± 2.1 2	0.001*
BAI score, mean ± SD		39.1 ± 13.4	32.8 ± 12.3 1	21.2 ± 11.5 2	< 0.01*
BDI score, mean ± SD		5.9 ± 4.0	6.7 ± 6.0	4.8 ± 3.1	0.452*
Attack severity (VAS), mean ± SD		7.8 ± 1.1	6.1 ± 1.1	−	< 0.01*
Attack frequency per month, mean ± SD		20.3 ± 7.9	22.6 ± 7.9	−	0.261*
Medication use per month, mean ± SD		30.2 ± 17.7	31.8 ± 18.9	−	0.782*
Disease duration, years, mean ± SD		12.9 ± 8.7	12.2 ± 11.0	−	0.317*

Abbreviations: BAI, Beck Anxiety Inventory; BDI, Beck Depression Inventory; PSQI, Pittsburgh Sleep Quality Index; SD, standard deviation; TTH, tension-type headaches; VAS, Visual Analogue Scale.

Note: *p* > 0.05.

The M-O group had considerably higher rates of family history of headache and VAS scores ( *p* < 0.05). No significant differences were observed among the patient groups in terms of medication use per month, attack frequency, and duration of illness ( *p* > 0.05). The PSQI scores exhibited notably higher values within both the M-O and TTH-O groups in contrast to the control group ( *p* < 0.01). The BAI score was considerably higher in the M-O group compared with both the TTH-O and control groups ( *p* < 0.01). In the TTH-O group, the BAI score was significantly higher compared with the control group ( *p* < 0.01, Table 1 ).

The total BIS score and the scores for the non-planning, motor, and attentional subgroups were significantly higher in both the M-O and TTH-O groups compared with the control group ( *p* < 0.01). No considerable differences were observed between the M-O and TTH-O groups in terms of total BIS score and scores for nonplanning, motor, and attentional subgroups ( *p* > 0.05), as shown in Table 2 .

**Table 2:** The BIS-11 scores in patient groups and healthy controls

BIS		^a^ Migraine	^b^ TTH	^c^ Control	*p* -value
Nonplanning	Mean ± SD	10.2 ± 3.3	9.4 ± 3.2	7.1 ± 2.9	***< 0.01*** ^K^
	Median	10.0	8.5	6.0 ^a,b^	
Motor	Mean ± SD	8.8 ± 2.2	8.6 ± 1.6	7.5 ± 2.2	***0.001*** ^K^
	Median	9.0	8.5	7.0 ^a,b^	
Attentional	Mean ± SD	11.4 ± 3.8	10.4 ± 3.0	7.4 ± 2.5	***< 0.01*** ^K^
	Median	12.0	11.0	6.0 ^a,b^	
Total	Mean ± SD	30.3 ± 7.4	28.3 ± 6.4	21.0 ± 6.3	***< 0.01*** ^K^
	Median	31.0	27.5	20.0 ^a,b^	

Abbreviations: BIS, Barratt Impulsiveness Scale; SD, standard deviation; TTH, tension-type headaches.

Notes: ^A^ ANOVA; ^K^ Kruskal-wallis (Mann-whitney u test) test; ^X2^ Chi-square test; ^a^ Difference with migraine group *p* < 0.05; ^b^ Difference with TTH group *p* < 0.05.

In the M-O group, a positive correlation was observed between the BIS non-planning score and the PSQI score (r = 0.389, *p* < 0.01), as well as between the non-planning score and attack frequency (r = 0.333, *p* = 0.02). Another positive correlation was detected between the BIS motor score and both the PSQI score (r = 0.389, *p* < 0.01), as well as attack frequency (r = 0.333, *p* = 0.02). A significant positive correlation was observed between the BIS attentional score and the PSQI score (r = 0.376, *p* = 0.01), BAI score (r = 0.386, *p* = 0.01), BDI score (r = 0.279, *p* = 0.05), and attack frequency (r = 0.376, *p* = 0.01). A notable positive correlation was observed between the total BIS score and the PSQI score (r = 0.455, *p* < 0.01), BAI score (r = 0.326, *p* = 0.03), and attack frequency (r = 0.452, *p* < 0.01), as shown in Table 3 .

**Table 3:** Correlation of BIS scores with other parameters in the migraine group

Parameters	BIS							
	Nonplanning		Motor		Attentional		Total	
	r	*p* -value	r	*p* -value	r	*p* -value	r	*p* -value
Age, years	-0.071	0.646	-0.201	0.191	0.066	0.672	-0.040	0.797
Schooling	-0.082	0.595	0.040	0.794	0.186	0.228	0.059	0.705
Attack severity (VAS)	-0.177	0.251	-0.074	0.633	0.065	0.674	-0.055	0.721
PSQI	0.389	**0.009**	0.392	**0.008**	0.376	**0.012**	0.455	**0.002**
BAI score	0.113	**0.464**	0.207	0.178	0.386	**0.010**	0.326	**0.031**
BDI score	-0.073	0.639	0.084	0.588	0.279	0.050	0.163	0.289
Attack frequency per month	0.333	**0.027**	0.379	**0.011**	0.376	**0.014**	0.452	**0.002**
Medication use per month	0.162	0.293	0.255	0.095	0.167	0.279	0.265	0.082
Disease duration	-0.188	0.221	-0.169	0.274	-0.002	0.991	-0.109	0.481

Abbreviations: BAS, Beck Anxiety Inventory; BDS, Beck Depression Inventory; BIS, Barratt Impulsiveness Scale; PSQI, Pittsburgh Sleep Quality Index; VAS, Visual Analogue Scale.

**Notes:** Spearman's correlation analysis; *p* > 0.05.

In regression analysis between the variables in the migraine group and the BIS, it was calculated that the nonplanning subdimension is influenced by the PSQI variable. An increase of one unit in the PSQI value will lead to an increase of 0.281 units in the nonplanning value. It is estimated that this is explained by the PSQI variable at a rate of 16%. The motor subdimension was found to be influenced by the variables age, PSQI, and medication use per month.

An increase of one unit in the age variable will result in a decrease of 0.054 units in the motor value. In contrast, an increase of one unit in the PSQI and medication use per month variables will increase the motor value by 0.146 and 0.051 units, respectively. It is calculated that the motor subdimension is explained by the existing independent variables at a rate of 30.3%. The Attentional subdimension was determined to be influenced by the PSQI variable. An increase of one unit in the PSQI value will result in an increase of 0.253 units in the attentional subdimension value. It is estimated that this subdimension is explained by the PSQI variable at a rate of 23.9%. The BIS was found to be influenced by the PSQI and medication use per month variables; an increase of one unit in these variables will increase the BIS value by 0.597 and 0.315 units, respectively. It is calculated that the scale is explained by the current independent variables at a rate of 32.4% ( Table 4 ).

**Table 4:** Multiple linear regression of BIS scores with other parameters in the migraine group

BIS	Variables	Coefficients		Model	
		B	*p* -value	Adj. R ^2^	*p* -value
Nonplanning	Constant	8.354	< 0.01	0.160	0.004
	PSQI	0.281	0.004		
Motor	Constant	8.231	< 0.01	0.303	0.001
	Age	-0.054	0.041		
	PSQI	0.146	0.016		
	Medication use month	0.051	0.003		
Attentional	Constant	4.613	0.026	0.239	0.003
	PSQI	0.253	0.026		
Total	Constant	19.983	< 0.01	0.324	< 0.01
	PSQI	0.597	0.005		
	Attack frequency	0.315	0.016		

**Abbreviations:** BIS, Barratt Impulsiveness Scale; PSQI, Pittsburgh Sleep QualityIndex.

Notes: *p* > 0.05; B, unstandardized regression coefficient; Adj. R, Adjusted R2.

In the TTH-O group, a notable negative correlation was observed between the BIS nonplanning score and schooling (r = -0.331, *p* = 0.04). A considerable positive correlation was observed between the BIS motor and VAS scores (r = 0.364, *p* = 0.02), as well as between the BIS motor and BDI scores (r = 0.342, *p* = 0.03) ( Table 5 ).

**Table 5:** Correlation of BIS scores with other parameters in the TTH group

Parameters	BIS							
	Nonplanning		Motor		Attentional		Total	
	r	*p-* value	r	*p-* value	r	p *-* value	r	*p-* value
Age, years	-0.100	0.552	-0.101	0.547	-0.299	0.068	-0.174	0.279
Schooling	-0.331	**0.042**	-0.052	0.756	-0.101	0.547	-0.279	0.090
Attack severity (VAS)	0.034	0.840	0.364	**0.025**	0.256	0.120	0.245	0.138
PSQI	0.164	0.325	0.083	0.621	0.290	0.078	0.250	0.130
BAI, mean ± SD	0.234	0.157	0.130	0.435	0.280	0.089	0.269	0.102
BDI, mean ± SD	0.053	0.754	0.342	**0.036**	0.033	0.843	0.124	0.459
Attack frequency per month	-0.066	0.693	0.168	0.312	0.162	0.332	0.041	0.805
Medication use per month	0.111	0.507	0.119	0.477	0.221	0.182	0.192	0.248
Disease duration, years	0.195	0.240	-0.061	0.716	-0.049	0.769	0.085	0.610

Abbreviations: BAI, Beck Anxiety Inventory; BDI, Beck Depression Inventory; BIS, Barratt Impulsiveness Scale; PSQI, Pittsburgh Sleep Quality Index; SD, standard deviation; TTH, tension-type headache; VAS, Visual Analog Scale.

Notes: *p* > 0.05. Spearman's correlation analysis.

In the regression analysis between the variables in the TTH-O group and the BIS, it was calculated that the nonplanning subdimension is influenced by the Schooling variable. An increase of one unit in the Schooling value will lead to a decrease of 0.335 units in the nonplanning value. It is estimated that the non-planning subdimension is explained by the Schooling variable at a rate of 15.4%.

The motor subdimension was found to be influenced by the BDI variable; an increase of one unit in this inventory will result in an increase of 0.093 units in the subdimension, at a rate of 10.0%.

The attentional subdimension was determined to be influenced by the age and BAI variables. An increase of one unit in the age variable will lead to a decrease of 0.098 units, and an increase of one unit in the BAI variable will result in an increase of 0.085 units in the subdimension, at a rate of 13.4%.

The BIS was found to be influenced by the age and schooling variables. An increase of one unit in the age and schooling variables will result in decreases of 0.198 and 0.579 units, respectively, in the BIS value. It is calculated that the BIS is explained by the current independent variables at a rate of 13.4% ( Table 6 ).

**Table 6:** Multiple linear regression of the BIS scores with other parameters in the TTH group

BIS	Variables	Coefficients		Model	
		B	*p-* value	Adj. R ^2^	*p-* value
Nonplanning	Constant	16.103	< 0.01	0.154	0.020
	Schooling	-0.335	0.007		
Motor	Constant	7.978	< 0.01	0.100	0.030
	BDI	0.093	0.030		
Attentional	Constant	11.759	< 0.01	0.145	0.025
	Age	-0.098	0.020		
	BAI	0.085	0.034		
Total	Constant	42.776	< 0.01	0.134	0.030
	Age	-0.198	0.036		
	Schooling	-0.579	0.020		

Abbreviations: BAI, Beck Anxiety Inventory; BDI, Beck Depression Inventory; BIS, Barratt Impulsiveness Scale; TTH, tension-type headache; B, unstandardized regression coefficient; Adj. R, Adjusted R2.

Notes: *p* > 0.05.

## DISCUSSION

In the present study, patients with M-O and TTH-O were compared with each other and with healthy controls in terms of impulsiveness. It was found that both groups were more impulsive compared with healthy controls. However, no difference in impulsiveness was observed between the M-O and TTH-O groups.

There are numerous studies on migraine and potential cognitive impairments, particularly focusing on impulsiveness and impulsive decision-making in migraine patients. 11 13 14 15 16 In a study of 155 subjects comparing impulsiveness among those with chronic migraines patients, MOH, and healthy individuals, no significant differences in impulsiveness were found. 13 In another study examining impulsiveness, migraine patients were divided into two groups based on the presence or absence of aura and compared with healthy controls. However, no significant results were observed. 14

A functional magnetic resonance imaging (MRI) study of the reward system in patients with chronic migraine with and without MOH found increased resting-state activity in the reward system among those with MOH compared with chronic migraine patients and healthy controls. Additionally, there was evidence of disrupted connectivity in this system among patients with MOH, leading to higher rates of impulsive choices in this group. 11

Cognitive impairments in TTH have been less studied, with limited data available on the topic. 12 Considering this headache type's high lifetime prevalence, identifying a specific risk of cognitive impairment would be a nonspecific approach. 12 24 Unlike migraine, there are results indicating that neither cognitive impairment nor decreased school performance during childhood were observed in individuals diagnosed with TTH in early adulthood. 25 Furthermore, it can be associated with higher rates of depression, anxiety, and sleep disturbances compared with healthy individuals. 26 A population-based study investigating the relationship between attention-deficit/hyperactivity disorder (ADHD) or its symptoms and primary headaches in children concluded that both migraine and TTH may be comorbid with hyperactive-impulsive behaviors. 25 To the best of our knowledge, no study in the literature has specifically focused on impulsiveness in TTH.

In the present study, the M-O and TTH-O groups showed similar levels of chronicity, with no differences in attack frequency or illness duration. Both groups exhibited higher levels of impulsiveness compared with healthy controls. On the other hand, the absence of differences in impulsiveness between chronic migraine and chronic TTH patients with MOH suggests the latter's association with impulsiveness, a shared subset of these two headache types.

Additionally, it is known that pain can cause attentional deficits. 27 Previous studies demonstrated that chronic headaches could impair attentional performance. 28 29 30 Attention is one of the most important cognitive functions of the frontal axis, and its deficiency can lead to disinhibition, potentially increasing impulsiveness. 31 32 In our study, a positive correlation between headache attack frequency and impulsiveness was found in both the migraine and TTH groups. The finding that motor impulsiveness increases as the VAS score increases in the TTH-O group supports the relationship between headache, attention, and impulsiveness. Furthermore, the results of the regression analysis suggest that the frequency of migraine attacks may influence impulsiveness, which could also be extrapolated to any pain attack.

It is known that sufficient and quality sleep is one of the most important factors influencing attention and impulse control. 33 34 In chronic headaches, particularly in migraines, the impact of more severe pain can also affect sleep quality. 35 36 Poor sleep can lead to impaired attention, which in turn can contribute to increased impulsiveness. In this study, all subdomains of impulsiveness, including attentional, motor, and nonplanning, increased as sleep quality deteriorated. The results of the regression analysis revealed that sleep quality influenced the total and subgroup impulsiveness scores in migraine patients, whereas no similar effect was observed in TTH.

Depression and anxiety are frequently comorbid with primary headaches, particularly migraine and TTH, occurring more often than would be expected by chance. 37 Both depression and anxiety have a negative impact on attention. 38 In this study, no considerable difference was found between the M-O and TTH-O groups in terms of depression and anxiety scores. However, it was found that as depression and anxiety increased in the M-O group, attention-related impulsiveness also increased. In the regression analysis, it was observed that in patients with TTH-O, depression affected motor impulsiveness, while the anxiety score influenced attentional impulsiveness.

Our study had some limitations. First, most of the patients were female. Considering the information that impulsiveness tends to have a higher prevalence in males, this predominance in our sample could introduce bias. Second, methodologically, it was considered that comparing headache groups with and without MOH could help better understand the differences between migraine and TTH. Third, only the number of patients could be obtained to reach the minimum required for power analysis. It was challenging to find patients with chronic TTH who had no psychiatric comorbidities and no accompanying migraine pain.

In conclusion, in this study we compared the impulsiveness of patients with chronic migraine and TTH accompanied by MOH with each other and with healthy controls, considering that excessive medication use could exhibit a pattern similar to drug abuse. In the M-O group, impulsiveness was found to correlate with both the frequency of migraine attacks and the number of medications used monthly. In the TTH-O group, depression was associated with motor and anxiety with attentional impulsiveness. Both groups exhibited greater impulsiveness compared with healthy controls, and no significant difference was observed between the M-O and TTH-O groups. It is hypothesized that MOH constitutes a shared cluster of impulsiveness in both primary headache groups. However, it should be noted that attention deficits are directly associated with impulsiveness, as well as being related to pain, poor sleep quality, depression, and anxiety, which can accompany chronic headaches.
